# Differential characteristics of vaginal versus endometrial microbiota in IVF patients

**DOI:** 10.1038/s41598-024-82466-9

**Published:** 2024-12-16

**Authors:** Alina Polifke, Annika von Schwedler, Rebecca Gulba, Ralf Bensmann, Alexander Dilthey, Najib N. R. Nassar, Patrick Finzer

**Affiliations:** 1 Institut für Medizinische Mikrobiologie und Krankenhaushygiene, Universitätsklinikum Düsseldorf, Universitätsstr. 1, 40225 Düsseldorf, Germany; 2 dus.ana, Düsseldorf Analytik, Immermannstrasse 65 A, 40210 Düsseldorf, Germany; 3 ZOTZ / KLIMAS MVZ Düsseldorf-Centrum GbR, Immermannstrasse 65A, 40210 Düsseldorf, Germany; 4 Novum Zentrum für Reproduktionsmedizin, Akazienallee 8 - 12, 45127 Essen, Germany

**Keywords:** Genital microbiota, Vaginal, Endometrial, IVF patients, RIF, RM, Medical research, Molecular medicine

## Abstract

Abnormal female reproductive tract microbiota are associated with gynecological disorders such as endometriosis or chronic endometritis and may affect reproductive outcomes. However, the differential diagnostic utility of the vaginal or the endometrial microbiome and the impact of important technical covariates such as the choice of hypervariable regions for 16 S rRNA sequencing remain to be characterized. The aim of this retrospective study was to compare vaginal and endometrial microbiomes in IVF patients diagnosed with implantation failure (IF) and/or recurrent pregnancy loss (RPL) and to investigate the overlap between established vaginal and endometrial microbiome classification schemes. An additional aim was to characterize to which extent the choice of V1-V2 or V2-V3 16 S rRNA sequencing schemes influences the characterization of genital microbiomes. We compared microbiome composition based on V1-V2 rRNA sequencing between matched vaginal smear and endometrial pipelle-obtained biopsy samples (*n* = 71); in a sub-group (*n* = 61), we carried out a comparison between V1-V2 and V2-V3 rRNA sequencing. Vaginal and endometrial microbiomes were found to be *Lactobacillus*-dominated in the majority of patients, with the most abundant *Lactobacillus* species typically shared between sample types of same patient. Endometrial microbiomes were found to be more diverse than vaginal microbiomes (average Shannon entropy = 1.89 v/s 0.75, *p* = 10^−5^) and bacterial species such as *Corynebacterium* sp., *Staphylococcus* sp., *Prevotella* sp. and *Propionibacterium* sp. were found to be enriched in the endometrial samples. The use of two widely used clinical classification schemes to detect microbiome dysbiosis in the reproductive tract often led to inconsistent results vaginal community state type (CST) IV, which is associated with bacterial vaginosis, was detected in 9.8% of patients; however, 31,0% of study participants had a non-Lactobacillus-dominated (NLD) endometrial microbiome associated with unfavorable reproductive outcomes. Results based on V2-V3 rRNA sequencing were generally consistent with V1-V2-based; differences were observed for a small number of species, e.g. Bifidobacterium sp., Propionibacterium sp. and Staphylococcus sp. and with respect to slightly increased detection rates of CST IV and NLD. Our study showed that endometrial microbiomes differ substantially from their vaginal counterparts, the application of a trans-cervical sampling method notwithstanding. Characterization of endometrial microbiomes may contribute to the improved detection of women with an unfavorable reproductive outcome prognosis in IVF patients..

**Supplementary Information:**

The online version contains supplementary material available at 10.1038/s41598-024-82466-9.

## Introduction

The female genital tract is colonized by a continuum of microbiota, ranging from the typically more diverse, but lower-biomass, bacterial communities in the endometrium to the typically less diverse, but higher-biomass, communities in the vagina^1^. In reproductive-age women, vaginal bacterial communities are mainly dominated by lactobacilli^2^; via production of lactic acid, secretion of active compounds, and immune modulation, lactobacilli contribute to hampering the growth of unfavorable or pathogenic bacteria in the vagina^3^. By contrast, the composition of the endometrial microbiome has remained less well-characterized; this is due to challenges associated with obtaining pure trans-cervical samples without contaminating vaginal material, potential biases associated with different types of sampling catheters used (e.g. pipelle, embryo-transfer-catheter etc.), and due to the fact that many studies of the endometrial microbiome have traditionally relied on culture-based methods to investigate the presence or absence of specific bacterial taxa^4,5^. However, with the more recent introduction of next-generation sequencing to the study of endometrial microbiomes, enabling the detection of non-culturable microorganisms and a more quantitative assessments of community structure, a more comprehensive picture of the composition of endometrial bacterial communities has begun to emerge^4^. Accordingly, the most reported state of the uterine microbiome in healthy women of reproductive age is dominated by lactobacilli; although other taxa are commonly detected, such as *Propionibacterium*,* Streptococcus*,* Bifidobacterium*,* Gardnerella* and *Prevotella*^6^.

Important gynecological disorders such as bacterial vaginosis (BV), endometriosis, and chronic endometritis (CE) are associated with changes in the microbial community structures of the reproductive tract^7^. For example, BV is associated with a loss of *Lactobacillus* dominance and the increase of mainly anaerobic bacteria such as *Gardnerella*,* Atopobium*,* Prevotella*,* Aerococcus* etc^8^. ; endometriosis has been associated with decreased *Lactobacillus* abundances and an increased presence of *Streptococcus* spp. and *Enterobacteriaceae*^9^; and in chronic endometritis (CE), disturbance in the *Lactobacillus* dominance and higher abundance non-*Lactobacillus* species such as *Gardnerella*,* Streptococcus* spp. and *Enterobacteriaceae*has been reported^9,10^. Well-established classification schemes for detecting genital microbiome dysbiosis include the Community State Type (CST) system for vaginal microbiota^2^ and the distinction between “*Lactobacillus*-dominated” (LD) and “non-*Lactobacillus*dominated” (NLD) endometrial microbiomes^11^. Within the CST system, CST IV is clinically associated with bacterial vaginosis and a lower vaginal pH; CST IV is defined by a loss of *Lactobacillus* dominance (relative *Lactobacillus* abundance < 50%) and often associated with increased abundances of *Gardnerella vaginalis* (CST IV-A), *together with Atopobium vaginae* (CST IV-B) or a diverse array of facultative and strictly anaerobic bacteria such as *Prevotella* spp., *Streptococcus* spp., *Enterococcus* spp., *Bifidobacterium* spp. or *Staphylococcus*spp. (CST IV-C)^12,13^). The other four CSTs, on the other hand, are *Lactobacillus*-dominated (≥ 50% abundance) and defined by the specific *Lactobacillus* species found to dominate (CST I is associated with *L. crispatus* (CST I); CST II, with *L. gasseri;* CST III, with *L. iners* (CST III); and CST V, with *L. jensenii*). For the LD/NLD classification scheme for endometrial microbiomes, NLD, which was associated with less favorable reproductive outcomes (see below), is defined as < 90% abundance of *Lactobacillus*sp^11^. Of note, the CST system and the LD/NLD scheme thus differ in their quantitative definition of *Lactobacillus* “dominance”.

The composition of reproductive tract microbiota also plays an important role in the context of the reproductive phenotypes, including receptivity and infertility due to recurrent pregnancy loss (RPL) or implantation failure (IF) to achieve pregnancy after repeated embryo transfer^6,14,15^. Low abundances or near-complete loss of lactobacilli in the vagina are linked to decreased rates of pregnancy after embryo transfer^16,17^. Similarly, lactobacilli were reported to be enriched in the endometrial microbiomes of women with live birth after IVF treatment and loss of *Lactobacillus*-dominance (i.e. NLD, see above) was associated with unsuccessful reproductive outcomes, including significant decreases of implantation, pregnancy, and live birth rates^11^; and miscarriages are associated with a lower abundance of *Lactobacillaceae*^18^.

The study of reproductive tract microbiomes can thus add an important dimension to the understanding of fertilization, implantation, and maintenance of pregnancy. While the role of the vaginal microbiota in reproduction has been studied extensively, however, the role of the endometrial microbiome, especially in the context of gynecological disorders and IVF, remains to be elucidated in a clinical routine setting. Important open questions remain with respect to the potential complementary diagnostic value of characterizing the endometrial microbiome, in addition to or even instead of the vaginal microbiome, and to which extent the results of the CST and LD/NLD genital microbiome classification schemes overlap. Furthermore, it is well-known that 16 S rRNA sequencing enables the characterization of genital microbiota and the distinction between the most common genital *Lactobacillus* species (*L. crispatus*,* L. iners*,* L. gasseri* and *L. jensenii;*^19^).

However, the studies carried out so far investigating the endometrial and vaginal microbiome have not investigated the species level of *Lactobacillus*, mostly sequencing the V3-V4, V4 or V3-V5 of the 16 S rRNA gene^11,20–25^. In contrast, interrogation of the V1-V3 regions of the 16 S rRNA gene has been successfully applied to characterize genital microbiota, enabling differentiating between the most common vaginal lactobacilli: *L. cirpsaturs*,* L. iners*,* L. gasseri* and *L. jensenii*^19^. 16 S rRNA sequencing typically comprises the joint amplification and sequencing of either the first and the second (V1-V2) or of the second and the third (V2-V3) hypervariable regions of the 16 S rRNA gene; the extent to which the choice of V1-V2 or V2-V3 influences measured genital microbiome compositions and the subsequent application of microbiome classification schemes, however, is not sufficiently well-characterized. The aim of this retrospective study was to address these open questions by comparing vaginal and endometrial microbiome structures in a cohort of women with implantation failure and recurrent pregnancy loss treated in an IVF outpatient clinic using a paired sampling approach and two 16 S sequencing approaches (V1-V2 and V2-V3).

## Results

### Patient cohort characteristics

**Fig. 1:**
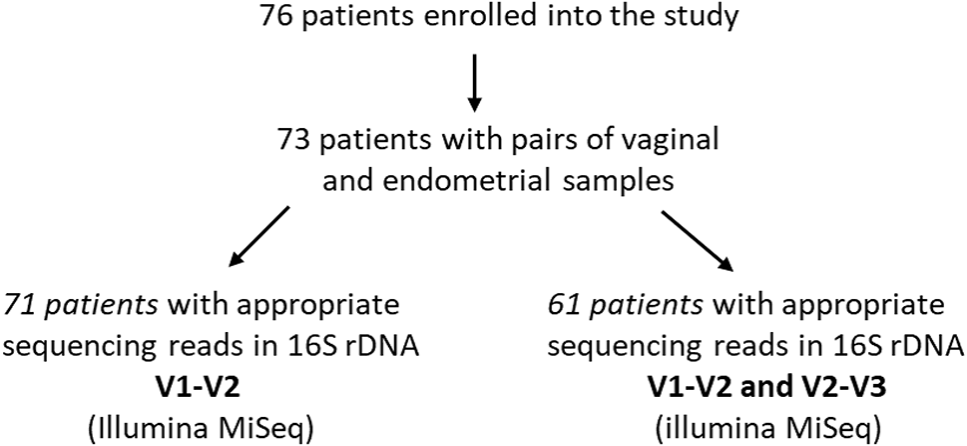
Overview of the study cohort. V1-V2, 16 S hypervariable regions 1 and 2; V2-V3, 16 S hypervariable regions 2 and 3.

**Fig. 2:**
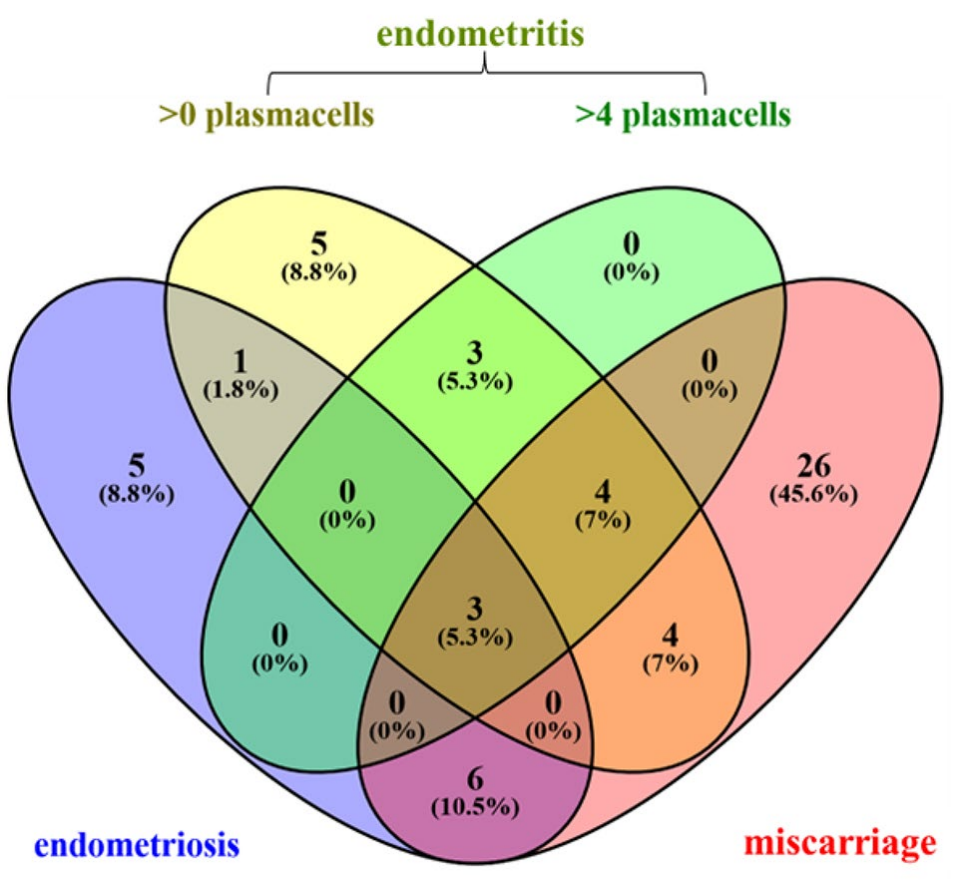
Multi-set Venn diagram showing unique and shared diagnoses across the study cohort (*n* = 71 patients).

73 patients with implantation failure (RIF) and/or recurrent pregnancy loss (RPL) provided paired vaginal and endometrial sample material and were included in the study (Fig. 1), exhibiting a mean age of 35 years (range 26–42) and a mean body mass index (BMI) of 24.5 (range 18.8 to 38.7; Table 1). Paired vaginal and endometrial sequence data were successfully generated for 71 patients who did not differ from the initial sample in terms of age and BMI. There were 1–2 implantation failures (IF) in 6 (8.4%) patients and 3 or more implantation failures (RIF) in 38 (53.5%) patients; pregnancy loss (PL) was present in 3 (4.2%) patients and recurrent pregnancy loss (RPL) in 19 (26.7%) patients. A combination of RIF and RPL was found in 4 (5.6%) patients. One patient was diagnosed with unexplained infertility. For 15 (21.1%) patients the diagnosis of endometriosis was noted in the medical reports and 39 study participants were found to have undergone past miscarriages. 10 (14.1%) and 20 (14.1%) patients, respectively, were diagnosed with chronic endometritis, depending on the threshold for the number of detected endometrial plasmacytes per high-power field (HPF). Some of the patients had overlapping diagnoses of endometritis, endometriosis and miscarriage (Fig. 2). The distribution of patients with regard to the indication for ART after miscarriage can be found in Suppl. Figure 4.

**Table 1:** Descriptive characteristics of the study population.

*n* = 73:		
Age (y)	35	(26–42)
BMI (kg/cm)	24,5	(18,8–38,7)
*n* = 71:		
Age (y)	35	(26–42)
BMI (kg/cm)	24,5	(18,8–38,7)
**Gynaecological Diagnosis**		
Chronic Endometritis	20*/71	28,2%
	10**/71	14,1%
Endometrioses	15/71	21,1%
**Reproductive History**		
Women report Live Birth	12/71	16,9%
Women report Miscarriage	39/71	54,9%
**Fertility Diagnosis** ^**1**^		
Implantation failure (IF)	06/71	8,4%
Repeated IF (RIF)	38/71	53,5%
Pregnancy loss (PL)	03/71	4,2%
Recurrent PL (RPL)	19/71	26,7%
RIF + RPL	04/71	5,6%
unexplained infertility	01/71	1,4%
**Reproductive Outcome** ^**2**^		
Pregnancy	34/71	47,9%
Live Birth	16/71	22,5%
Miscarriage	10/71	14,1%

# 73 patients with pairs of vaginal and endometrial samples. ## 71 patients with appropriate sequencing reads in 16 S rDNA V1-V2. Diagnosis based on + more than 1 plasma cell (PC) / high power field (HPF) or ** more than 4 PC / HPF.

^1^Diagnosis according to the medical records. IF: 1–2 failed embryo transfers (ET) attempts; RIF: three or more failed ET attempts; PL: loss of one pregnancy; RPL: loss of two or more pregnancies.

^2^Reproductive outcomes in June 2022 (see methods).

### Comparison of vaginal and endometrial microbiota based on 16 S V1-V2 rRNA sequencing

Paired vaginal and endometrial V1-V2 16 S rRNA sequencing data were successfully generated for 71 patients, yielding an average number of 340,835 (range: 58,938–1,255,987) sequencing reads per vaginal sample and an average number of 230,883 (range: 15,718–3,046,106) sequencing reads per endometrial sample. Mean reads in OTUs per vaginal and endometrial sample were 256,560 and 141,328, respectively. Two patients were excluded because the generated number of reads (in the vaginal sample of the first patient and in the endometrial sample of the second patient) fell below the minimum read threshold.

**Fig. 3:**
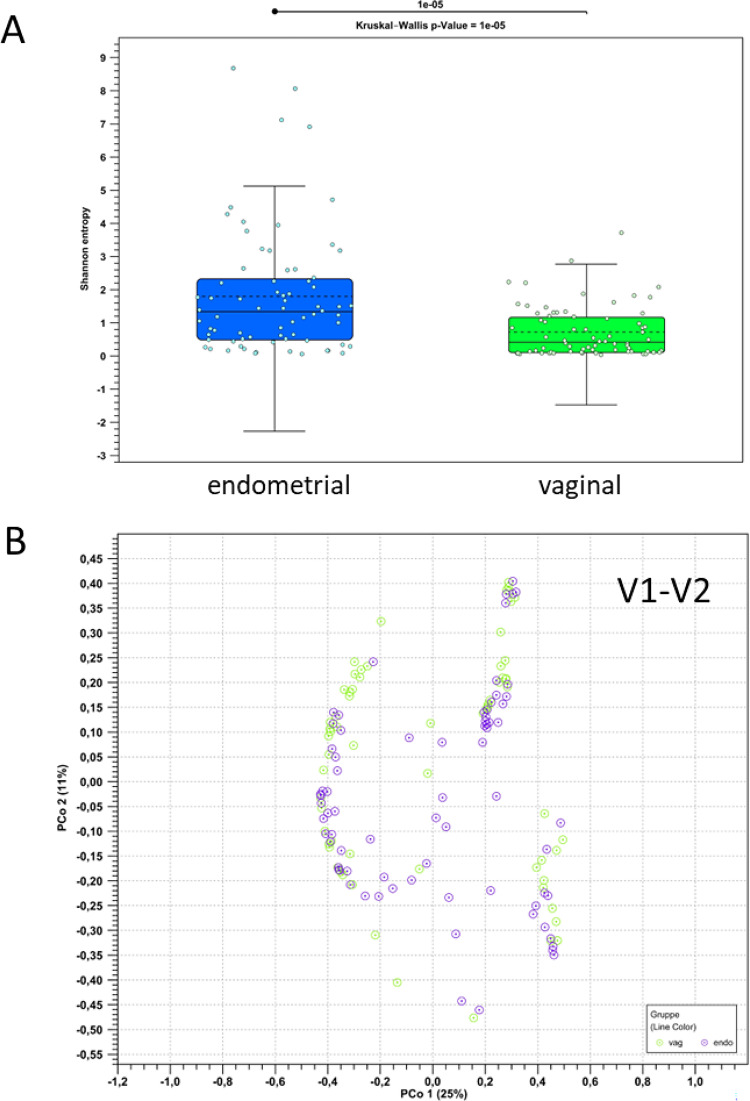
Shannon diversity (**A**) and PCoA visualization (**B**) of vaginal and endometrial microbiome compositions for *n* = 71 patients, based on V1-V2 16 S rRNA sequencing. The reported p-value for comparing the diversity between vaginal and endometrial samples was calculated using the Kruskal-Wallis test.

Based on the generated V1-V2 16 S rRNA sequencing data, we carried out a comparison of vaginal and endometrial microbiomes. First, we compared the overall diversity of the vaginal and the endometrial samples (Fig. 3A). Rarefaction analysis showed that alpha diversity (Shannon entropy) reached a stable plateau in all samples (data not shown), indicating that comparison of diversity metrics between different groups could be performed. At an average Shannon entropy of 1.89 (range: 0.04 to 9.04), the characterized endometrial samples were found to be significantly more diverse than the characterized vaginal samples, which exhibited an average Shannon entropy of 0.75 (range: 0.01 to 3.71; Kruskal-Wallis p-value: 10^−5^).

Second, we carried out a Principal Coordinates Analysis (PCoA; Fig. 3B). In this analysis we found that the distributions of vaginal and endometrial samples in PCoA spaces largely overlap, consistent with similar large-scale community structures (see below), although there is increased diversity in endometrial samples.

**Fig. 4:**
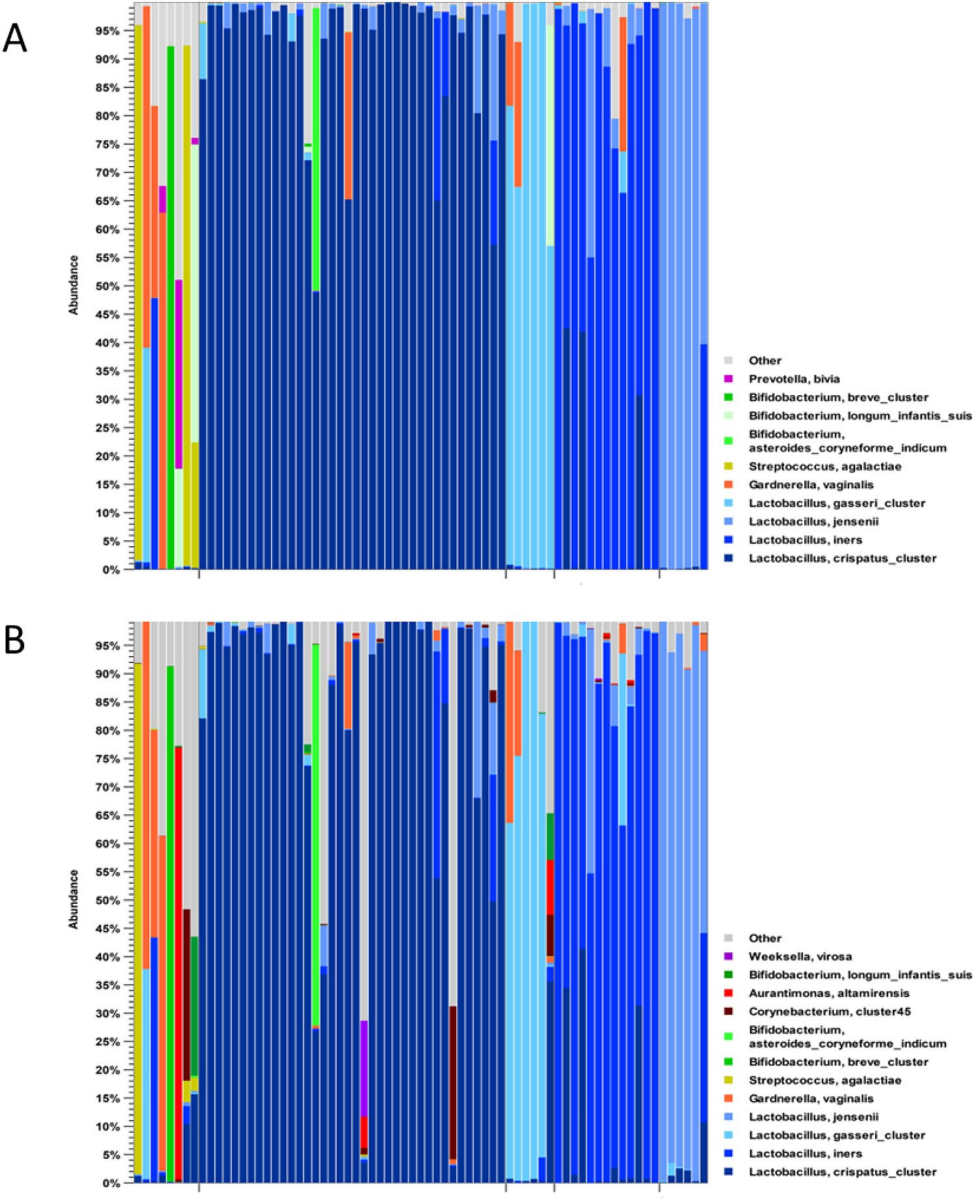
Per-patient taxonomic composition of vaginal (**A**) and endometrial (**B**) samples from *n* = 71 patients, based on V1-V2 16 S rRNA sequencing. Vaginal and endometrial samples from the same patients are aligned vertically.

Third, we compared the vaginal and endometrial microbiome compositions in individual patients (Fig. 4). As expected, in most patients, both vaginal and endometrial microbiomes were dominated by lactobacilli; specifically, non-dominance by lactobacilli (here defined as *Lactobacillus* abundance < 50%) in the vaginal and endometrial sample was observed in 8 patients, and non-dominancy by lactobacilli in only the endometrial sample was found in an additional 5 patients (Fig. 4).

**Fig. 5:**
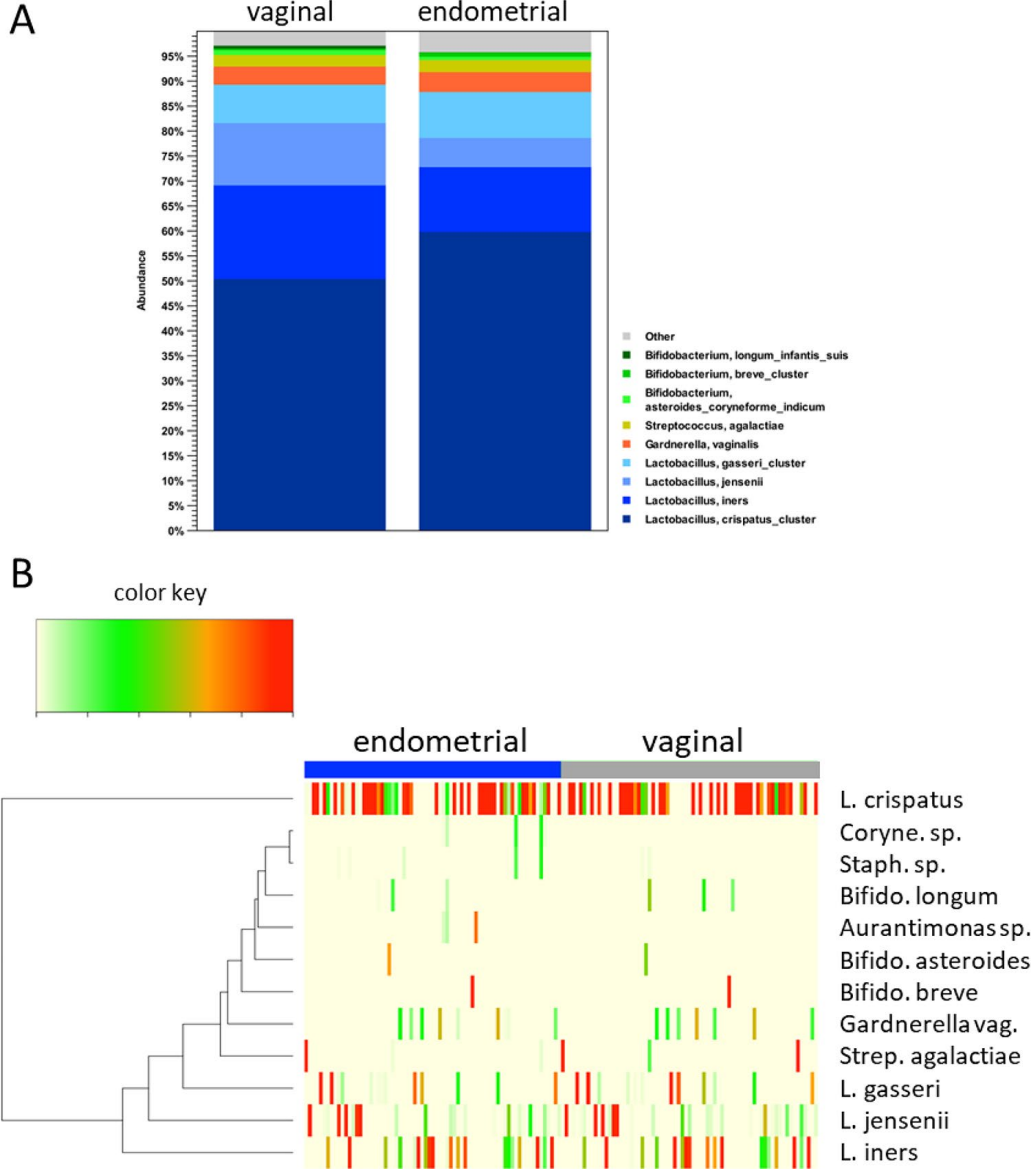

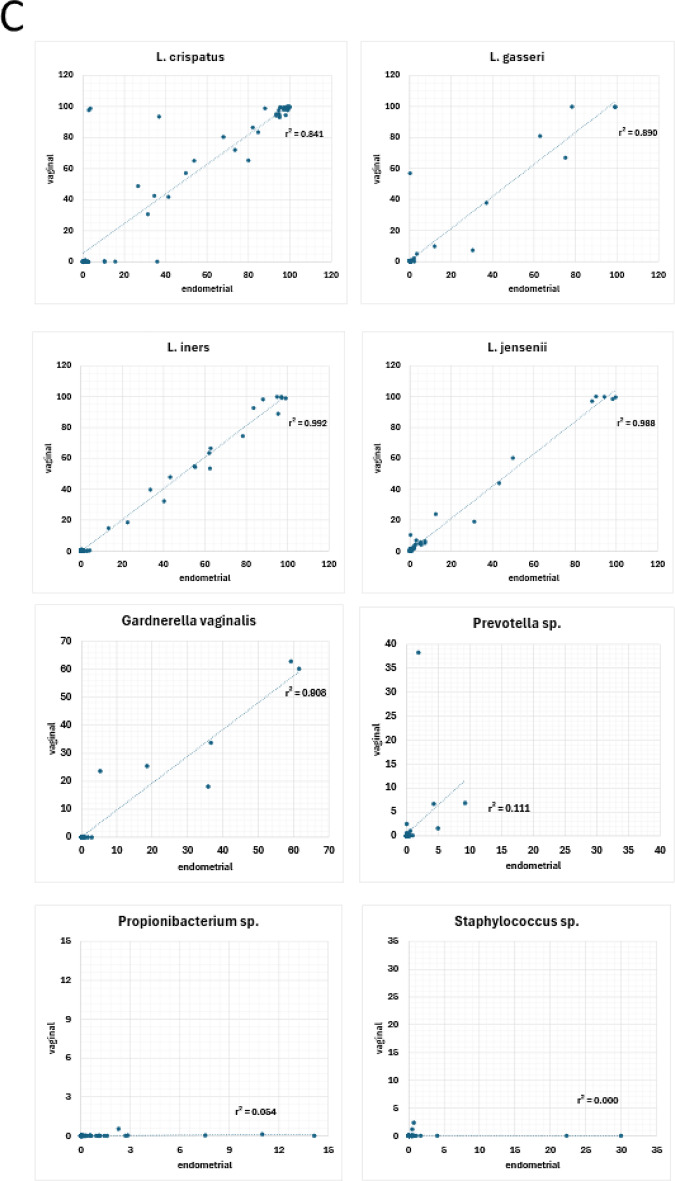
Abundances of specific bacterial species in vaginal and endometrial samples from *n* = 71 patients, based on V1-V2 16 S rRNA sequencing. (**A**) Bar charts showing the mean values of the most abundant operational taxonomic units / bacterial species in vaginal and endometrial samples. (**B**) Heatmap representing operational taxonomic units / bacterial species in vaginal and endometrial samples. The rows show bacterial species, the columns represent subjects. L.: *Lactobacillus*, Coryne.: *Corynebacterium*, Staph.: *Staphylococcus*, Bifido.: *Bifidobacterium*, Strep.: *Streptococcus*. (**C**) Within-patient comparison and correlations of vaginal and endometrial abundances for specific bacterial species.

Furthermore, in the large majority of investigated vaginal-endometrial sample pairs from the same patient, the majority *Lactobacillus* species was shared between the two sample types (Figs. 4 and 5A and B; for the genus or family level see Suppl. Figures 1 and 2 ). The correlation (Pearson’s r²) between vaginal and endometrial relative abundances ranged from 0.842 to 0.993, with the paired showing no difference in mean abundances (*p* > 0.05) for the four *Lactobacillus* species most relevant in the genital microbiome context. Vaginal and endometrial relative abundances were also found to be strongly correlated for *Gardnerella* sp. (r^2^ = 0.908; *p* = 0.697), *Atopobium* sp. (r^2^ = 0.974; *p* = 0.253), *Sneathia* sp. (r^2^ = 0.999; *p* = 0.320), *Bifidobacterium* sp. (r^2^ = 0.849; *p* = 0.224) and *Streptococcus* sp. (r^2^ = 0.517; *p* = 0.148) (Fig. 5C, Supplementary Table 1). By contrast, lower correlations were observed for *Propionibacterium* sp. (r^2^ = 0.054; *p* = 0.007, paired t-test), *Staphylococcus* sp. (r^2^ = 0.000; *p* = 0.088), *Prevotella* sp. (r^2^ = 0.119; *p* = 0.359) and *Corynebacterium* sp. (r^2^ = 0.024; *p* = 0.043) (Fig. 5C and Supplementary Table 1). In general, non-*Lactobacillus* species detected vaginally were almost always also detected in the endometrial sample from the same patient. By contrast, non-*Lactobacillus* species were detected in the endometrial microbiome but not in the vaginal sample from the same patient, indicating an increased abundance of these species in the endometrial microbiome at least in a subset of patient. This emphasizes the polymicrobial nature of the endometrial microbiome.

### Established vaginal and endometrial classification schemes yield non-concordant results

To investigate the extent of overlap between established indicators of dysbiosis of the vaginal (CST IV) and endometrial (NLD) microbiomes, we carried out a CST and LD/NLD classification based on the generated V1-V2 16 S rRNA sequencing data. Results of the CST and LD/NLD classification of vaginal vs. endometrial microbiome samples are shown in Table 2.

**Table 2:** Vaginal vs. endometrial classification schemas.

		Vaginal (Community State Typs (CSTs))						
		**I**	**II**	**III**	**IV-A**	**IV-B**	**IV-C**	**V**
**Endometrial**	**LD**	31 (43,7%)	2 (2,8%)	9 (12,7%)	0	0	0	7 (9,8%)
	**NLD**	7 (9,9%)	4 (5,6%)	4 (5,6%)	0	2 (2,8%)	5 (7,0%)	0
	**total (%)**	38 (53,5%)	6 (8,5%)	13 (18,3%)	0	2 (2,8%)	5 (7,0%)	7 (9,8%)

Endometrial and vaginal samples from 71 patients sequenced with V1-V2 16s rRNA.

CST: community state types according to VALENCIA (https://github.com/ravel-lab/VALENCIA), LD: *Lactobacillus* Dominance (> 90), NLD: Non-*Lactobacillus* Dominance (< 90).

**Fig. 6:**
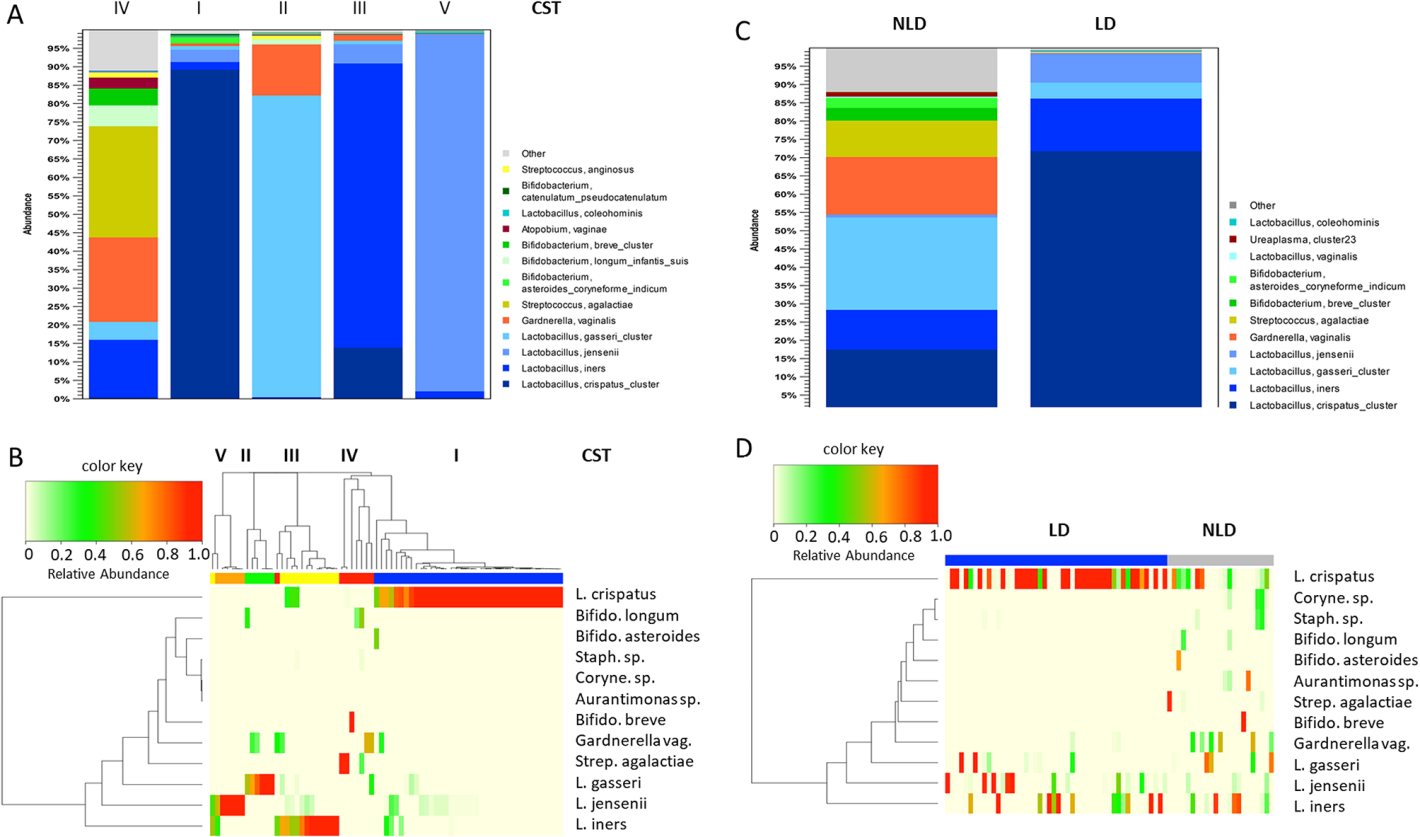
Mean abundances of specific bacterial taxa in vaginal (**A**) and endometrial (**C**) and heatmaps representing operational taxonomic units / bacterial species in vaginal (**B**) and endometrial (**D**) samples; the rows show bacterial species, the columns represent subjects. L.: *Lactobacillus*, Coryne. *Corynebacterium*, Staph.: *Staphylococcus*, Bifido.: *Bifidobacterium*, Strep.: *Streptococcus*. Samples from *n* = 71 patients, grouped according to CST (https://github.com/ravel-lab/VALENCIA) and LD/NLD (Moreno et al., 2016) classification and based on V1-V2 16 S rRNA sequencing.

The majority of vaginal samples were classified as CST I (53.5%); 18.3% of vaginal samples were classified as CST III; 9.8% as CST V and, at 8.5% CST II showed the lowest relative frequencies. Only 9.8% of the included vaginal samples were classified as CST IV, characterized by *Lactobacillus* abundance < 50%. The distribution of CSTs in our study was comparable to CSTs in a group of 97 European-ancestry patients published by Ravel et al.^2^. At the cumulative level, *Streptococcus* and *Gardnerella* accounted for a large fraction of overall abundances within the samples classified as CST IV (Fig. 6A and B); increased abundances of *Gardnerella* were also found in CST II.

By comparison, 31,0% of endometrial microbiome samples were classified as NLD; while all patients with CST IV vaginal microbiomes were also found to exhibit an NLD endometrial microbiome, NLD endometrial microbiomes were also found in patients with CSTs I, II and III. Established indicators of dysbiosis of the vaginal and endometrial microbiomes are therefore not redundant. At the cumulative level, the group of NLD endometrial microbiomes exhibited increased abundances of non-*Lactobacillus* species (Fig. 6C and D) - above 5% abundance of *G. vaginalis* in 27,3%, of *Streptococcaceae* in 9,1% and of *Bifidobacteria* in 22,7% of NLD patients.

### Classification of vaginal and endometrial microbiomes remains non-concordant after adjustment

To investigate whether the classification of vaginal and endometrial microbiomes remained non-concordant after adjustment, we applied the following modifications to the established classification approaches. First, we applied the LD/NLD criterion to the vaginal microbiomes in our study and investigated overlap with LD/ND endometrial results (Table 3). 22.5% of vaginal samples were classified as NLD, representing a substantial increase over the proportion of vaginal microbiomes classified as CST IV; however, the proportion of NLD in vaginal microbiomes remained lower than in endometrial microbiomes (absolute difference: 10%). Second, Haahr et al.^17^ found that increased diversity of the vaginal microbiome (threshold: Shannon diversity index ≥ 0.93) was linked to poor reproductive outcomes; we applied this threshold in our study and found that the overall percent of vaginal microbiomes falling above this diversity threshold was comparable to the rate of NLD endometrial microbiomes. Third, we applied the above threshold to the vaginal and endometrial samples and found an even greater difference between the two, reflecting the higher diversity of the endometrial material. Fourth, we also applied the CST classification scheme to all sample types in our study (Supplementary Table 4) and found a higher, though non-perfect, degree of overlap between vaginal and endometrial samples, classifying, e.g. 2 samples with vaginal CSTs I and II as endometrial CST IV. Therefore, the classification schemes, with the potential exception of the CST scheme, remained substantially non-concordant at the per-individual level (Table 3; Supplementary Table 4). While the investigated adjustments thus led to an increased overlap between endometrial and vaginal classification results, substantial differences remained, indicating differences and the non-redundancy of microbial communities along the genital tract.

**Table 3:** Classification of genital microbiota along reproducitve outcome schemas.

A	Lactobacillus abundance above vs. below 90% in vaginal vs. endometrial samples.			
	*Chi² < 0.0001*	**Endometrial**		
		LD (n)	NDL (n)	total (n)
	**Vaginal**			
	LD (n)	48 (67,6%)	7 (9,9%)	55 (77,5%)
	NLD (n)	1 (1,4%)	15 (21,1%)	16 (22,5%)
	total (n)	49 (69,0%)	22 (31,0%)	71 (100%)
**B**	**Vaginal diversity (Shannon entropy) vs. endometrial lactobacillus dominance**			
	*Chi² < 0.0001*	**Endometrial**		
		LD (n)	NLD (n)	total (n)
	**Vaginal**			
	< 0.93* (n)	41 (57,7%)	7 (9,9%)	48 (67,6%)
	>= 0.93* (n)	8 (11,3%)	15 (21,1%)	23 (32,4%)
	total (n)	49 (69,0%)	22 (31,0%)	71 (100%)
**C**	**Vaginal vs. endometrial diversity (Shannon entropy)**			
	*Chi² = 0.0002*	**Endometrial**		
		< 0.93* (n)	>= 0.93* (n)	total (n)
	**Vaginal**			
	< 0.93* (n)	21 (29,6%)	27 (38,0%)	48 (67,6%)
	>= 0.93* (n)	0	23 (32,4%)	23 (32,4%)
	total (n)	21 (29,6%)	50 (70,4%)	71 (100%)

Endometrial and vaginal samples from 71 patients sequenced with V1-V2 16s rRNA. LD: *Lactobacillus* Dominance (> 90), NLD: Non-*Lactobacillus* Dominance (< 90). *Shannon entropy.

### Results of V2-V3 16s rRNA sequencing are largely, though not completely, consistent with V1-V2 16 S rRNA sequencing

To investigate to which extent the choice of targeting the V1-V2 or V2-V3 hypervariable regions for 16 S rRNA sequencing influences vaginal and endometrial microbiome community analysis, we generated V2-V3-based 16 S rRNA sequencing data in a sub-group of 61 patients (Fig. 1). Baseline metrics of the investigated sub-group such as weight (mean BMI: 24.5; range: 18.8–38.7) and age (mean age: 35 years; range: 26–42) were comparable to these of the main study cohort. Overall V2-V3 sequencing read counts for the vaginal (average read count: 297,962; range, 67,491–997,682) and endometrial (average read count: 198,830; range, 26,358–905,102) samples were similar to those observed in the V1-V2-based analysis; the number of reads in OTUs, however, was generally lower in the V2-V3 data than in the V1-V2 data.

**Fig. 7:**
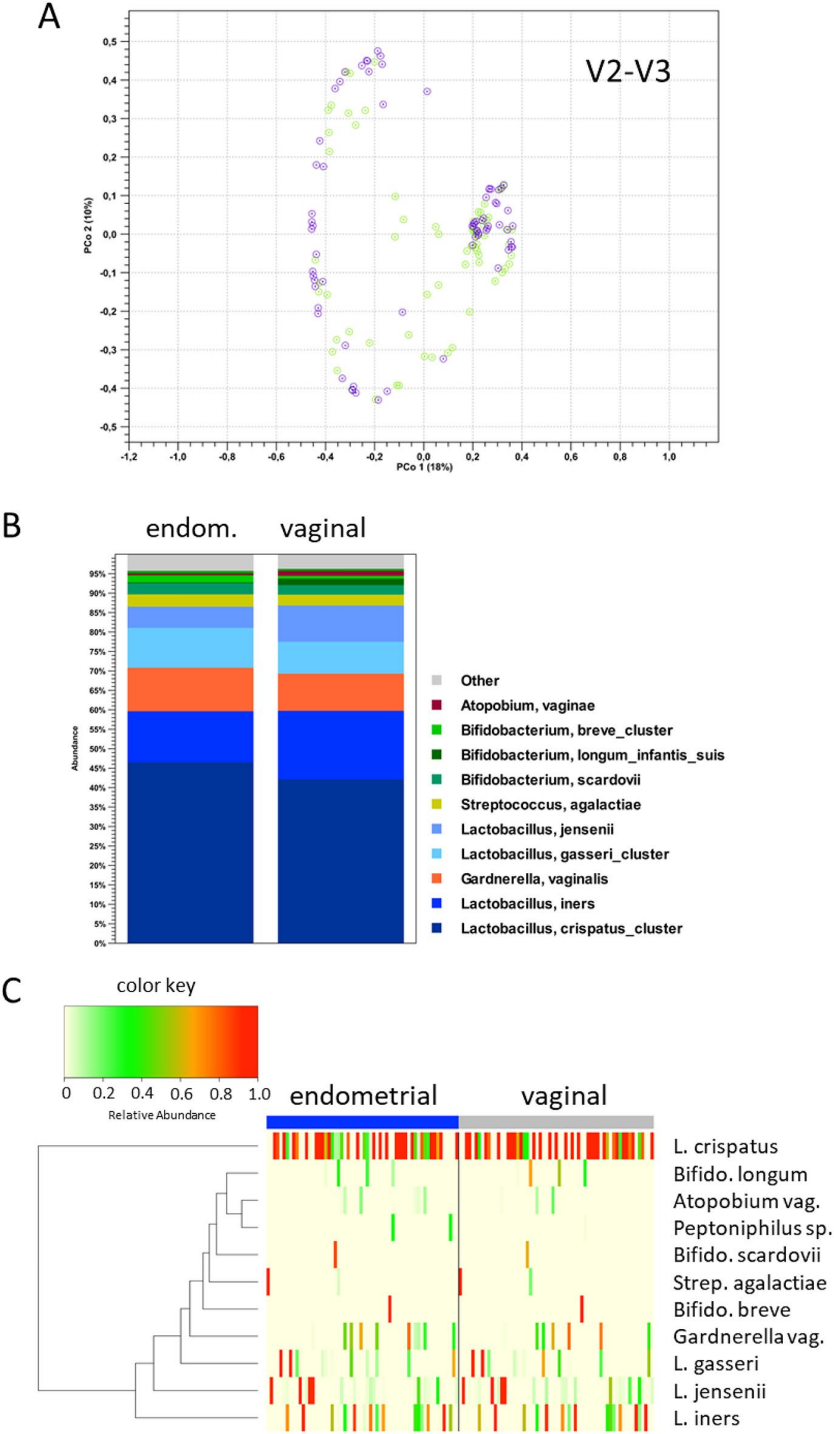
(**A**) PCoA visualization of vaginal (green) and endometrial (blue) microbiome compositions. (**B**) Mean abundances of specific bacterial species in vaginal and endometrial samples. (**C**) Heatmap representing operational taxonomic units / bacterial species in vaginal and endometrial samples; the rows show bacterial species, the columns represent subjects. L.: *Lactobacillus*, Coryne.: *Corynebacterium*, Staph.: *Staphylococcus*, Bifido.: *Bifidobacterium*. Analyses based on V2-V3 16 S rRNA sequencing (*n* = 61 patients).

**Fig. 8:**
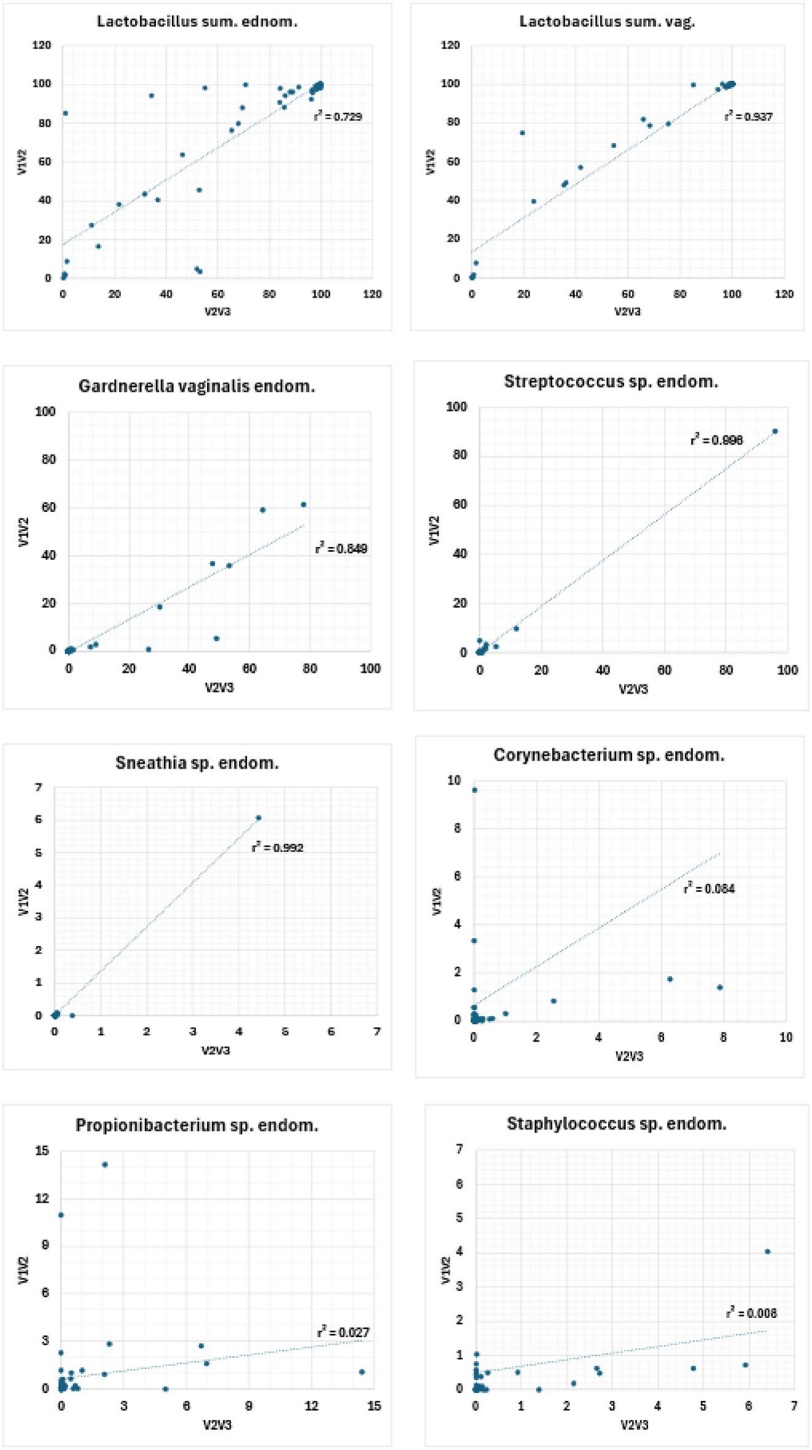
Comparison of the relative abundances of specific bacterial taxa based on V1-V2 and V2-V3 16 S rDNA sequencing for vaginal (vag.) and endometrial (endom.) samples. *Lactobacillus* sum. includes *L. crispatus*,* L. gasseri*,* L. iners* and *L. jensenii*.

First, we carried out a PCoA of the V2-V3 data (Fig. 7A) and found that it recapitulated the results of the V1-V2-based PCoA (Fig. 3B), indicating that the characteristics of both microbiota are stable properties of the respective communities and independent of the hypervariable regions used for sequencing. Second, we carried out a comparison at the level of individual bacterial taxa; the cumulative distribution of the V2-V3 data (Fig. 7B and C) was similar to that observed for the V1-V2 data (Fig. 5A) and high correlations (r² ≥ 0.8 and t-test *p* > 0.05, except for *L. crispatusp* = 0.000) Fig. 8 and Supplementary Table 2) between V2-V3- and V1-V2-based abundances in the endometrial and vaginal samples were observed for lactobacilli and other species such as *Streptococcus* sp. (endo.: r^2^ = 0.996; *p* = 0.718, vag. r^2^ = 0.978; *p* = 0.384), Atopobium sp. (endo.: r^2^ = 0.694; *p* = 0.0519, vag.: r^2^ = 0.993; *p* = 0.109) and *Sneathia* sp (endo.: r^2^ = 0.992; *p* = 0.467, vag.: r^2^ = 1.000; *p* = 0.317), as well as in the endometrial samples only for *Gardnerella vaginalis* (endo.: r^2^ = 0.849; *p* = 0.012).

Substantially lower correlations, however, were observed at both sample sides for *Propionibacterium* sp. (endo.: r^2^ = 0.027; *p* = 0.988, vag.: r^2^ = 0.454; *p* = 0.522) and *Bifidobacterium sp.* (endo.: r^2^ = 0.966; *p* = 0.016, vag.: r^2^ = 0.979; *p* = 0.017), as well as endometrial for *Corynebacterium* sp. (r^2^ = 0.084; *p* = 0.304) and *Staphylococcus* sp. (r^2^ = 0.008; *p* = 0.765) and vaginal for *Gardnerella vaginalis* (vag.: r^2^ = 0.851; *p* = 0.069) (Fig. 8, Supplementary Fig. 5, Supplementary Table 2), demonstrating that the choice of hypervariable regions targeted for 16 S rRNA sequencing can influence the abundance estimates of individual species. Third, we compared the results of CST and LD/NLD between the V1-V2 and V2-V3 (Tables 4 and 5). While overall classification results were found to be similar, the number of vaginal samples classified as CST IV, and the number of endometrial samples classified as NLD, was increased based on the V2-V3 dataset, indicating that V2-V3 rRNA sequencing may exhibit higher sensitivity for the detection of dysbiotic genital microbiome community states. Importantly, however, the observed differences between V1-V2 and V2-V3 16 S rRNA sequencing did not affect the observation of non-redundancy between vaginal and endometrial classification schemes.

**Table 4:** Microbiota classification using V1-V2 or V2-V3 primers for 16 S sequencing.

V1-V2						
	Vaginal (Community state types (CSTs))					
	I	II	III	IV	V	Total (%)
**Endometrial**						
**LD**	28 (45.9%)	2 (3.3%)	8 (13.1%)	0 (0.0%)	5 (8.2%)	43 (70.5%)
**NLD**	6 (9.8%)	3 (4.9%)	3 (4.9%)	6 (9.8%)	0 (0.0%)	18 (29.5%)
**total (%)**	34 (55.7%)	5 (8.2%)	11 (18.0%)	6 (9.8%)	5 (8.2%)	61 (100%)

**V2-V3**						
	**Vaginal (Community state types (CSTs))**					
	**I**	**II**	**III**	**IV**	**V**	**Total (%)**
**Endometrial**						
**LD**	24 (39.3%)	2 (3.3%)	5 (8.2%)	0 (0.0%)	4 (6.6%)	35 (57,4%)
**NLD**	9 (14.8%)	2 (3.3%)	4 (6.6%)	10 (16.4%)	1 (1.6%)	26 (42.6%)
**total (%)**	33 (54.1%)	4 (6.6%)	9 (14.8%)	10 (16.4%)	5 (8.2%)	61 (100%)

61 patients with appropriate reads in 16 S rDNA V1-V2 and V2-V3. CST: community state types according to VALENCIA (https://github.com/ravel-lab/VALENCIA), LD: *Lactobacillus* Dominance (> 90), NLD: Non-*Lactobacillus* Dominance (< 90).

**Table 5:** Comparison of CST classification for V1-V2 and V2-V3.

	CST	V1V2					
		I	II	III	IV	V	Total
**V2V3**	**I**	33					33
	**II**		4				4
	**III**			9			9
	**IV**	1	1	2	6		10
	**V**					5	5
	**total**	34	5	11	6	5	61

61 patients with appropriate reads in 16 S rDNA V1-V2 and V2-V3. CST: community state types according to VALENCIA (https://github.com/ravel-lab/VALENCIA).

## Discussion

The aim of this retrospective study was to carry out a comparison between vaginal and endometrial microbiomes in a group of infertile patients treated in an IVF outpatient clinic and to investigate the overlap between established vaginal and endometrial microbiome classification schemes. An additional aim was to characterize to which extent the choice of V1-V2 or V2-V3 16 S rRNA sequencing schemes influences the characterization of genital microbiomes.

While our study confirmed a degree of similarity between endometrial and vaginal microbial communities – for example with respect to overall dominance by lactobacilli in the majority of analyzed vaginal and endometrial samples and substantial correlations between vaginal and endometrial abundances of many bacterial taxa in individual patients – it also confirmed fundamental differences between the two microbiomes. First, and in line with the literature^26^, endometrial microbiomes were found to be significantly more diverse. Second, low-abundance species such as *Propionibacterium* sp., *Prevotella* sp., *Staphylococcus* sp. or *Corynebacterium* sp. contribute to the increased diversity of the endometrial microbiome, and the detected differences in microbiome structure could be interpreted as an increased amount of biological information being encoded in the endometrial microbiome when compared to the vaginal microbiome. Third, established clinical microbiome classification schemes – the CST and LD/NLD schemes – yield non-overlapping results in a substantial number of cases, further underlining the differences between the two microbial communities and pointing towards the potential diagnostic utility of the endometrial microbiome.

One important limitation of our study is the employed trans-cervical sampling scheme, which may – cleaning of the portion prior to taking the endometrial biopsy notwithstanding – involve the contamination of the collected endometrial sample with microorganisms from the higher-biomass environments of the vagina or the cervical canal^1^. While such contamination may explain a degree of the similarities between endometrial and vaginal samples, the detection of lower-abundance species in the endometrial samples clearly demonstrates accessibility of the endometrial microbiome using the employed trans-cervical sampling scheme. In addition, we note that trans-cervical sampling schemes like the one employed in our study are suitable for implementation in clinical routine settings, increasing the relevance of our findings with respect to the potential development of clinical endometrial microbiome biomarkers. In addition, while it cannot be ruled out that some of the species exclusively found in the endometrial microbiome may represent false-positive detections driven by low biomass, the fact that we consistently observe many of these species across many samples in our study, and the fact that the same species are also found in other studies^11,27^, makes a high rate of false-positive detections seem less likely. For example, Moreno et al.^27^ detects the species we found in endometrial fluid and biopsy and identifies them as “nodes” of a bacterial network associated with reproductive outcomes.

We carried out a comparison between V1-V2 and V2-V3 16 S rRNA sequencing with respect to the characterization of vaginal and endometrial microbiomes and found that results based on the two approaches were largely consistent, though differences were observed with respect to specific bacterial taxa and with respect to the detection rates of CST IV and NLD. Specifically, our results indicated that sequencing of the V2-V3 region may exhibit higher sensitivity for dysbiotic community states and with respect to the detection of species like *Gardnerella vaginalis*. Consistent with this, while our study was not designed to detect novel outcome-associated biomarkers, when testing for the association between ≥ 10% abundance of *Gardnerella vaginalis*and pregnancy loss in an exploratory analysis, a lower p-value was observed based on V2-V3-based abundances than for V1-V2-based abundances (0.02 compared to 0.06; Chi-Squared test of independence). For gut microbiome analysis it has been shown that the choice of 16 S rRNA hypervariable regions – V3-V4 compared to V4-V5 – can have a substantial effect^28^. Studies focusing on the endometrial microbiome have often targeted varying hypervariable regions of the 16 S rRNA gene, e.g. V3-V5 or V4-V5^9,11,26^. However, using the V3-V5 hypervariable region of the 16 S rRNA gene resulted in the identification of the same bacterial OTUs in vaginal samples^11^, supporting the use of V1-V2 – and, by extension based on the results presented here, V2-V3 – approaches for the sequencing of endometrial biopsies. Future studies may pursue alternative or combined amplification approaches, or shotgun metagenomics. Possible future options, importantly, include (i) the possibility to target multiple hypervariable regions^27^and (ii) full-length 16 S rRNA sequencing approaches^29^. It is important to note, however, that full-length 16 S rRNA amplification approaches can also exhibit relevant biases with respect to the characterization of important members of genital microbiomes^29,30^.

Our study cohort was not selected with respect to medical history, diagnosis or treatment. In addition, we also considered different characteristics or subgroups for both patient groups - implantation failure and miscarriage: 1–2 implantation failures (IF) versus 3 or more (RIF) or 1 pregnancy loss (PL) versus 2 or more (RPL) while broadly representative of real-world patient characteristics encountered in the context of outpatient treatment for infertility; heterogeneity in the analyzed population presents challenges with respect to the definition of appropriate subgroups for downstream statistical analysis. We did therefore not attempt to correlate microbiome features with reproductive outcomes or to characterize covariates of microbiome composition; this is an important aim for future studies. Our findings are, however, in line with previous reports of genital microbiome dysbiosis in up to 40% of patients who failed IVF treatment^16,31,32^; about one third of our study group was found to exhibit NLD in the endometrial microbiome, which is known to be correlated with an unfavorable reproductive outcome^11^. In their study, Iwami et al.^33^ found an NLD proportion of approx. 23% in a group of 131 RIF patients, which roughly corresponds to the order of magnitude described by us. This means that, on the one hand, lower *Lactobacillus*abundances are found in the group of patients with RIF, but also in RPL^20^, but on the other hand, this only leads to a loss of *Lactobacillus*abundance below 90% (NLD) in a subgroup. While direct association between different CSTs and the reproductive outcome has not been demonstrated so far^17^, the clinical connection between bacterial vaginosis (BV) and infertility is well established^15,34^, and BV is associated with CST IV, found in approximately 10% of our patients. Interestingly, the *L. iners*-associated CST III has also been reported to be associated with decreased fertility^35^; CST III was found in another 18,3% of our study cohort. Of note, the classification of patients into CST IV vaginal and NLD endometrial microbiomes, community states associated with dysbiosis, did often not overlap in our study population. The observed differences persisted after application of our adjustment approaches, corroborating the distinct nature of the two microbiomes and highlighting the possibility that endometrial microbiome structures linked with unfavorable reproductive outcomes may also be found in patients with otherwise normal vaginal microbiota.

It is important to emphasize that none of the established classification schemes (CST or LD/NLD) is able to reliably predict unfavourable reproductive outcomes on its own; while the patients included in our study varied in their assignment according to these schemes, almost all with IF or RPL and a large proportion had some form of endometriosis. What is more, whether a classification scheme capable of predicting unfavorable reproductive outcomes can be developed based solely on the abundance of single or multiple *Lactobacillus* species – or even vaginal or endometrial microbiome community composition in general – remains open at this stage. It is possible that the accuracy of such a scheme could be increased by adding additional species, increasing the resolution of microbiome characterization approaches (see above), or by adding further clinical parameters.

In summary, our study confirmed the distinct nature of the endometrial microbiome in this cohort of patients and the general applicability of two widely used 16 S rRNA sequencing approaches for characterizing both vaginal and endometrial microbiomes. Our findings are consistent with the hypothesis that the biological information carried by the endometrial microbiome is complementary to the vaginal microbiome and that the endometrial microbiome may represent an important target of diagnostic and potentially therapeutic approaches in the field of reproduction medicine. The joint effect of the vaginal and endometrial microbiota on human reproductive outcomes warrants improved characterization in future studies.

## Methods

### Study design and study population

The study was conducted at “novum, Zentrum für Reproduktionsmedizin”, Essen / Duisburg, Germany. Between February 2020 and March 2021, 76 patients with implantation failure - and/or recurrent pregnancy loss (RPL)) gave written and informed consent to use their clinical data and sequencing analysis for a retrospective study in the lab the microbiota of endometrial biopsies were sequenced, together with their corresponding vaginal smear as controls: for 73 patients, corresponding vaginal swab and endometrial biopsy samples could be obtained. Information about pregnancy or delivery was extracted from medical records or obtained by telephone or email interviews conducted through June 2022. This study was approved by the Ethics Committee of the Faculty of Medicine at Heinrich Heine University Düsseldorf (Study: 2021 − 1448 – “MikrobiomART”) and all methods were carried out in accordance with the relevant guidelines and regulations.

### Clinical examination and sample collection

Vaginal and endometrial samples were taken at the mid luteal phase (between day 20 and 22 of the menstrual cycle). During the procedure, the external genitals were inspected and a transvaginal ultrasound (B-mode) was performed. Afterwards, the vaginal speculum was inserted and vaginal fluid was collected from the dorsal fornix. Subsequently, the portio was cleaned with octenidindihydrochlorid / phenoxyethanol (octenisept^®^) and the Pipelle catheter was introduced through the cervical canal into the uterine cavity, as described elsewhere^11^. The catheter contents (samples) were transferred directly into a 2 ml eNAT tube (Copan, Italy) and shipped at room temperature. The samples were stored at −20 degrees until DNA extraction.

### DNA extraction

Vaginal and endometrial samples were treated identically for DNA extraction. After thawing, samples were shortly vortexed and 250 µl sample volume was used for DNA extraction. DNA extraction was performed using the Power Fecal Pro DNA kit (Qiagen, Hilden, Germany), following the manufacturer´s protocol using the QIAsymphony extraction device (Qiagen, Hilden, Germany). DNA concentration was assessed using the UCP probe PCR kit (Qiagen, Hilden, Germany). An extraction blank was performed as a control to monitor for potential contamination.

### 16 S ribosomal RNA gene amplification and sequencing

Library preparation of the V1-V2 and V2-V3 regions of the 16 S rRNA was performed according to the manufacturer’s protocol using the QIAseq 16 S/ITS screening and regional panels (Qiagen, Hilden, Germany). DNA quantification was carried out using the Qubit 2.0 Fluorometer and the Qubit dsDNA HS Assay (Thermo Fisher Scientific, MA, USA). DNA fragment size was analyzed using the High Sensitivity D1000 kit (Agilent Technologies, Santa Clara, USA). Purified libraries were normalized and finally sequenced on the MiSeq system (Illumina Inc. San Diego, CA, USA) using v3 chemistry. A mock microbial community was used as a positive control for library preparation and sequencing. Image analysis and base calling were carried out directly on the MiSeq. Pairs of vaginal and endometrial samples from the same patient were always analyzed in parallel and within the same library preparation and sequencing run.

### Sequencing-based microbiome analysis and data analysis

Demultiplexing and sequencing data analysis, including the computation of sample composition, were carried out using the CLC Microbial Genomic Workbench 12.0.3 (Qiagen, Hilden, Germany). Raw read filtering and clustering into Operational Taxonomic Units (OTUs), followed by the elimination of low-frequency OTUs, were carried out at 97% pair-wise identity and using standard parameters. OTUs were labeled by mapping against the VM database and SILVA^36^.

### Statistical analysis

Further statistical analyses were carried out using the CLC Microbial Genomic Workbench 12.0.3 (Qiagen, Hilden, Germany), comprising the following steps: (i) Computation of alpha diversities (Shannon entropy^37^) for assessing biodiversity; (ii) Principal Coordinates Analysis (PCoA with Bray Curtis) for assessing microbial community structure; (iii) data visualization using box plots and scatter plots, and assessment of statistical significance based on the Kruskal-Wallis test. Pearson correlations and the coefficient of determination (r^2^) of the linear trend lines, as well as paired t-Tests were calculated using Excel. Correlations with an r^2^ value above 0.8 were defined as “highly correlating”, r^2^ values between 0.5 and 0.8 as “correlating” and values below 0.5 as “non-correlating”.

## Electronic supplementary material

Below is the link to the electronic supplementary material.

Supplementary Material 1

## Data Availability

The sequencing data are available at SRA under BioProject Identifier: PRJNA1095707 (https://urldefense.com/v3/__https://www.ncbi.nlm.nih.gov/bioproject/PRJNA1095707__;!!NLFGqXoFfo8MMQ! r36Zlgo1DzpZTyItW1QAMgP4ZiLMUoFHnnueNDR6Xrf96nzUu-1jJJ__CUK9vfKD06TvT2otRAxrhWKUgxLhWl24cvL3Qg$) / AccNr. SRP499453. Additional metadata are available upon reasonable request from the corresponding author.
