# Supplementary material for: Differential characteristics of vaginal versus endometrial microbiota in IVF patients
Source: Sci Rep. 2024 Dec 16;14:30508. doi: 10.1038/s41598-024-82466-9 (PMC11649765; doi:10.1038/s41598-024-82466-9)
Supplement: Supplementary file 1 — Supplementary Material 1 [file 41598_2024_82466_MOESM1_ESM.pdf]

Suppl. Fig. 1

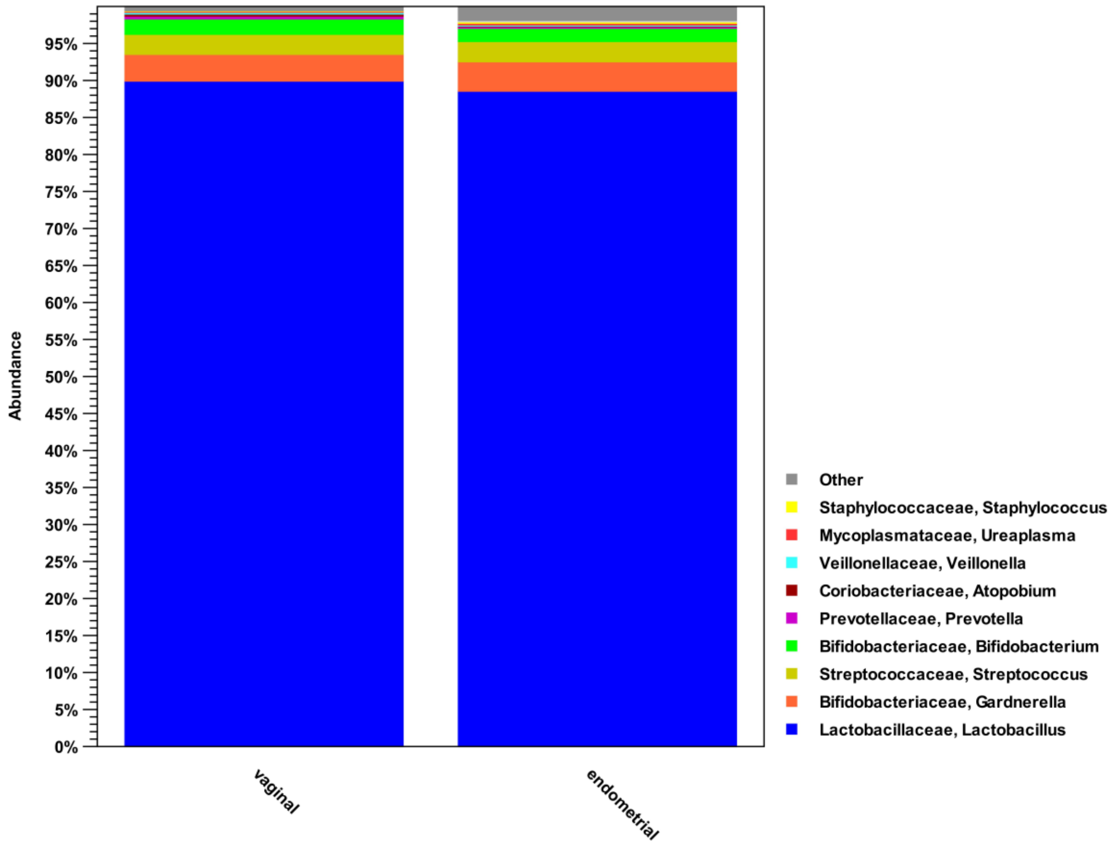

Suppl. Figure 1. Abundances of specific bacteria in vaginal and endometrial samples from n = 71 patients, based on V1-V2 16S rDNA sequencing. Bar charts showing the mean values of the most abundant operational taxonomic units at genus level in vaginal and endometrial samples.

Suppl. Fig. 2

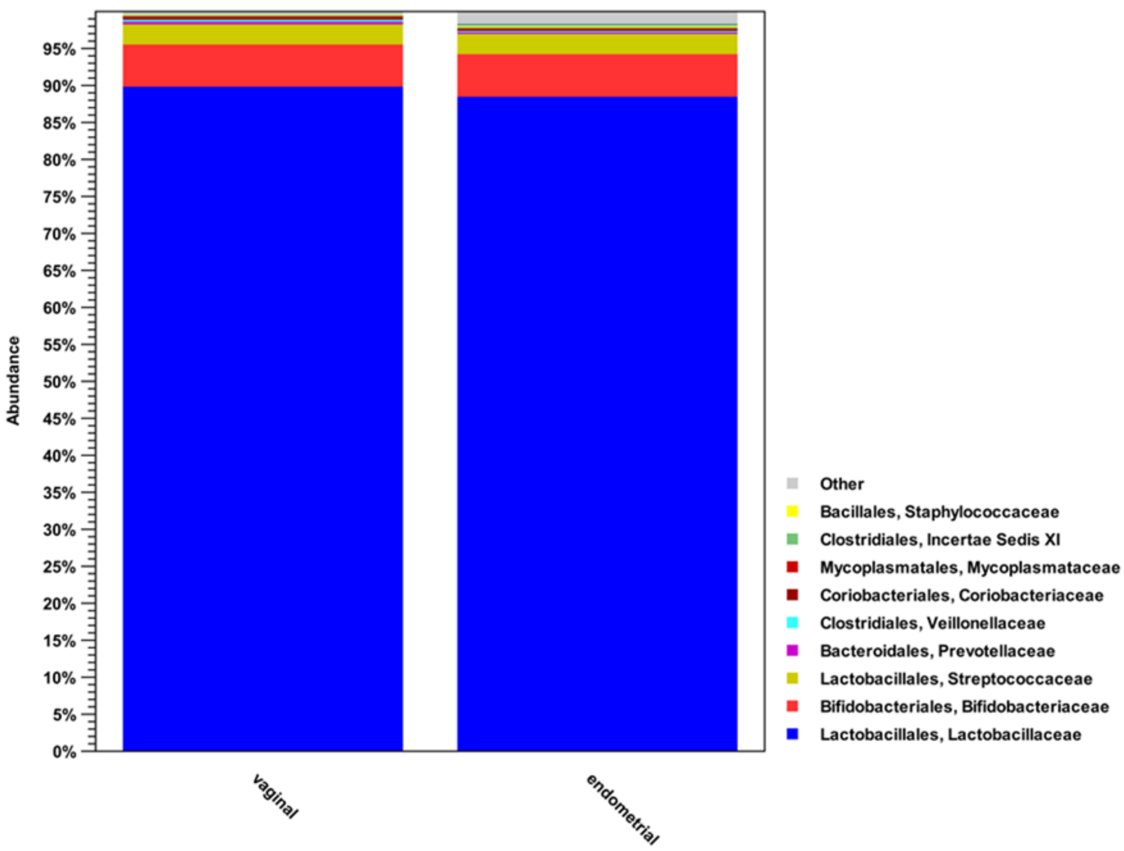

Suppl. Figure 2. Abundances of specific bacteria in vaginal and endometrial samples from n = 71 patients, based on V1-V2 16S rDNA sequencing. Bar charts showing the mean values of the most abundant operational taxonomic units at family level in vaginal and endometrial samples.

Suppl. Fig. 3

ZymoBIOMICS®
Microbial Community Standard

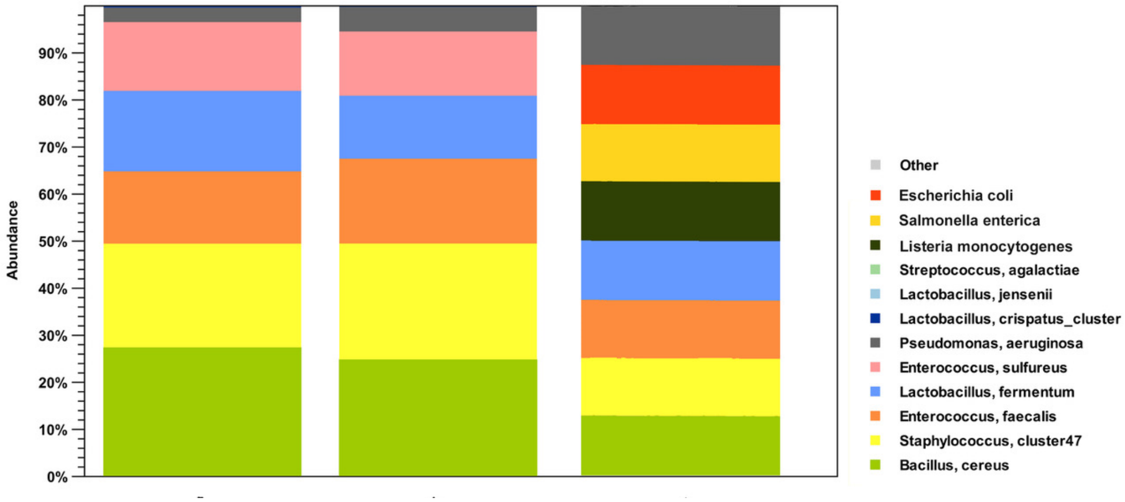

V1-V2 V2-V3 theoretical distribution

Abundances of specific bacterial taxa in ZymoBIOMICS microbial community standard by sequencing V1-V2 or V2-V3 16S rRNA region and theoretical distribution.

Suppl. Fig. 4

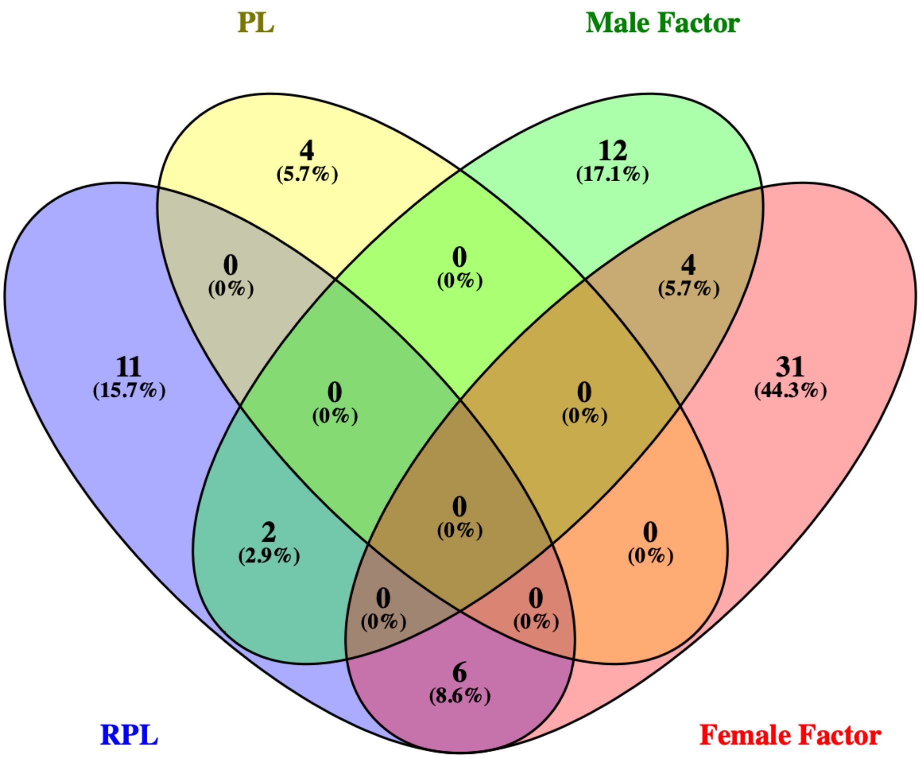

Distribution of patients regarding indications for ART after miscarriage. PL: pregnancy loss; RPL: recurrent pregnancy loss. In vitro fertilization (IVF) was performed in cases of female factor or idiopathic infertility (Female Factor); Intracytoplasmic Sperm Injection (ICSI) was performed in cases of sever male factor with/without combination with female factor, such as tubal factor etc. (Male Factor).

Suppl. Fig. 5

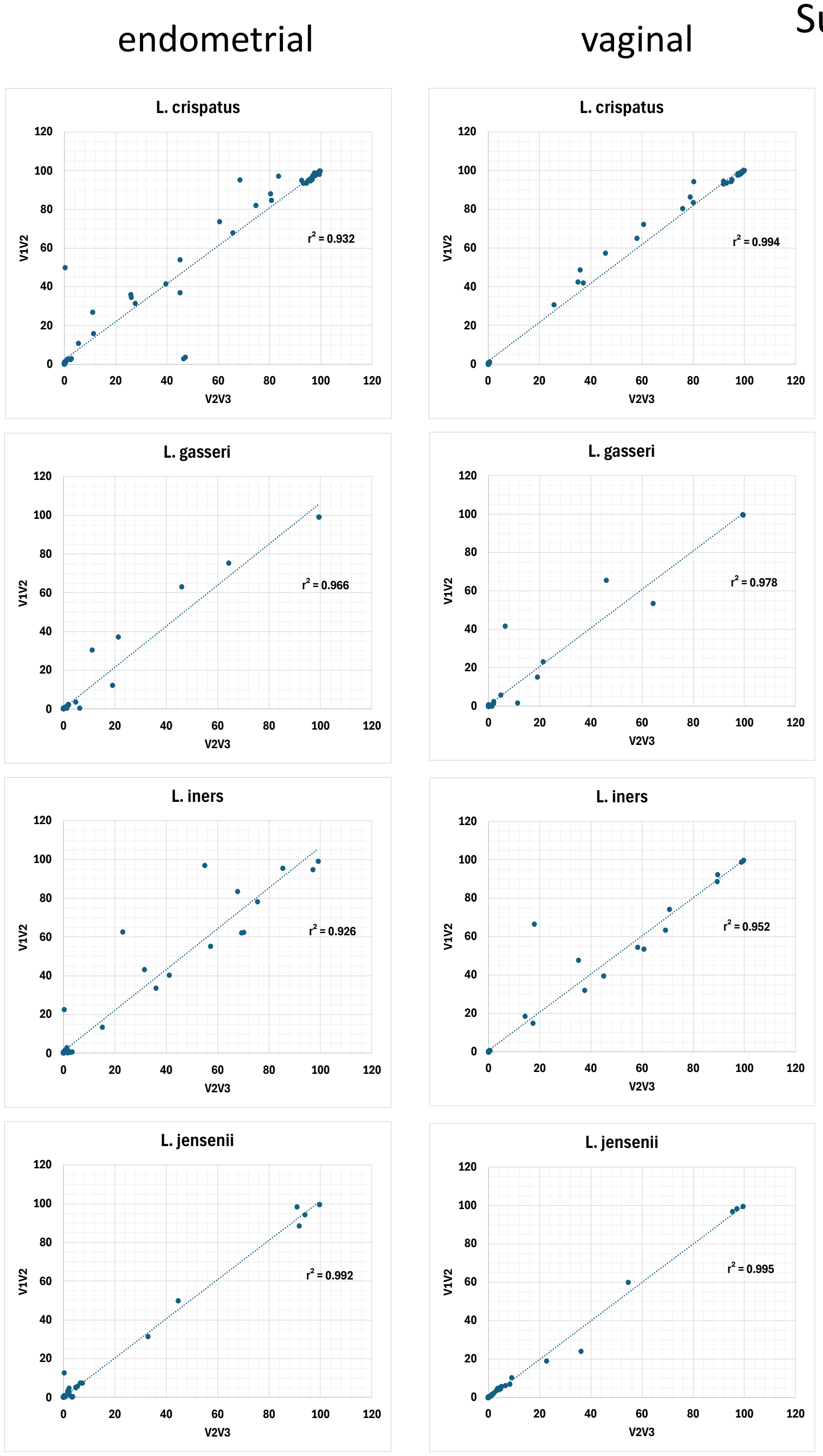
endometrial
vaginal
L. crispatus
r² = 0.932
L. crispatus
r² = 0.994
L. gasseri
r² = 0.966
L. gasseri
r² = 0.978
L. iners
r² = 0.926
L. iners
r² = 0.952
L. jensenii
r² = 0.992
L. jensenii
r² = 0.995
V1V2
V2V3
0
20
40
60
80
100
120

Suppl. Fig. 5

endometrial

vaginal

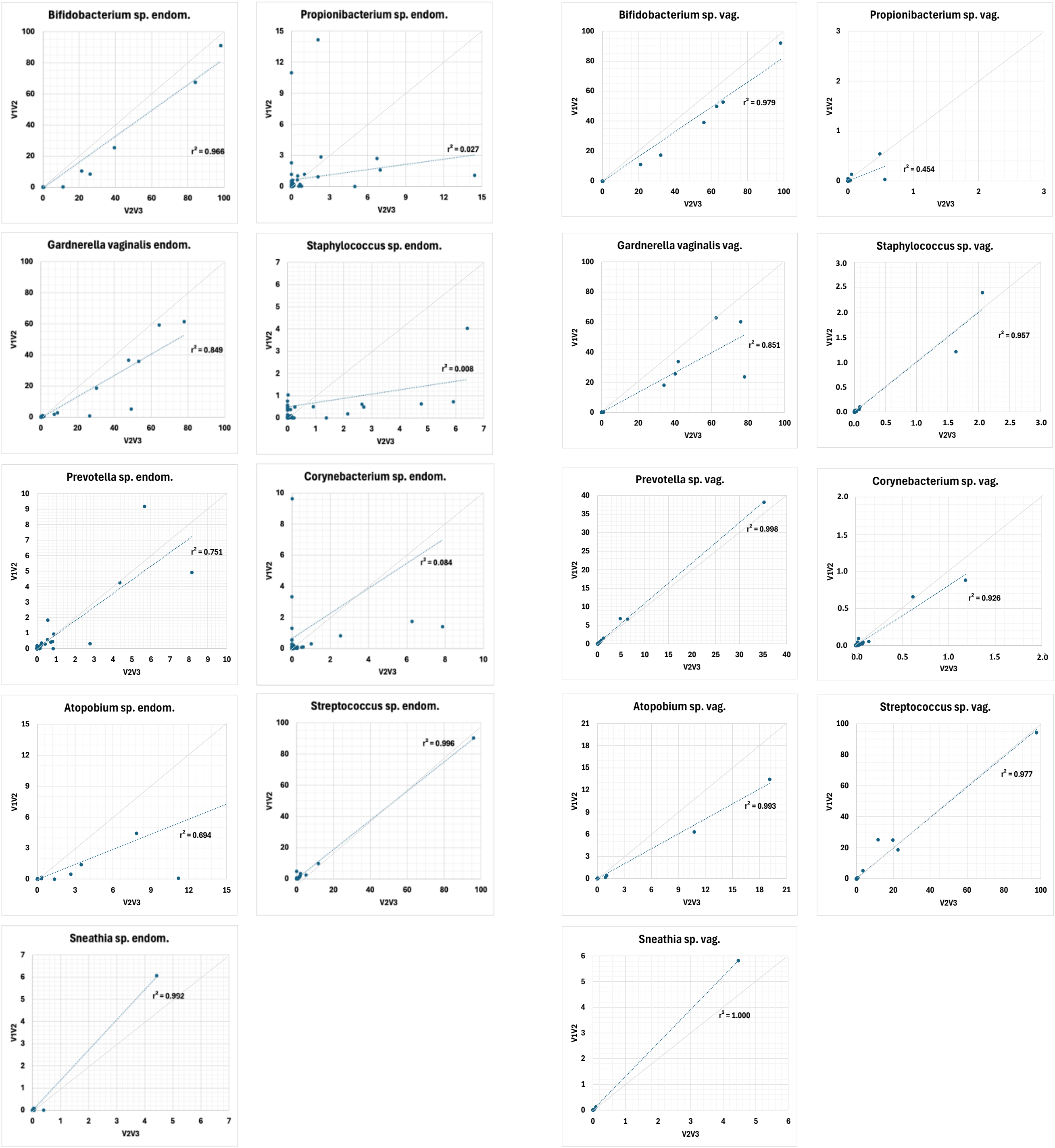

Suppl. Fig. 5

Comparison of the relative abundances of specific bacterial taxa based on V1-V2 and V2-V3 16S rRNA sequencing for vaginal and endometrial samples.

Supp. Table 1: Vaginal-endometrial correlation of abundance for specific bacterial species (n=71)

V1V2

A: Pearson correlation and paired t-Test

	L. crispatus	L. gasseri	L. iners	L. jensenii
Pearson´s r² =	0.8428	0.8901	0.9928	0.9878
t-Test p =	0.1332	0.3789	0.9218	0.0852

B: Perason correaltion and paired t-Test

	Corynebacterium	Bifidobacterium	Gardnerella v.	Atopobium v.	Prevotella sp.	Staphylococcus	Streptococcus	Propionibacterium	Sneathia sp.
Pearson´s r² =	0.0236	0.8487	0.9085	0.9742	0.1189	0.0002	0.5172	0.0536	0.9998
t-Test p =	0.0426	0.22432	0.69716	0.25352	0.35887	0.08807	0.148	0.00673	0.32048

Suppl. Table 2: Vaginal-endometrial correlation of abundance for different bacteria and primer-combination (n=61)

V1V2 / V2V3

A: Pearson correlation and paired t-Test

		L. crispatus	L. gasseri	L. iners	L. jensenii
endo. V1V2 vs V2V3	Pearson´s r^2 =	0.9316	0.9658	0.9259	0.9923
	t-Test p =	0.3090	0.1558	0.0869	0.2903
vag. V1V2 vs V2V3	Pearson´s r^2 =	0.9943	0.9783	0.9523	0.9947
	t-Test p =	0.0004	0.0556	0.4317	0.6980

B: Pearson correlation and paired t-Test

		Corynebacterium sp.	Bifidobacterium sp.	Gardnerella vaginalis	Atopobium sp.	Prevotella sp.	Staphylococcus sp.	Streptococcus sp.	Propionibacterium sp.	Sneathia sp.
endo. V1V2 vs V2V3	Pearson´s r^2 =	0.0838	0.9657	0.8486	0.6942	0.7513	0.0081	0.9957	0.0275	0.9919
	t-Test p =	0.3036	0.0164	0.0124	0.0519	0.7350	0.7650	0.7177	0.9885	0.4673
vag. V1V2 vs V2V3	Pearson´s r^2 =	0.9625	0.9790	0.8511	0.9933	0.9982	0.9575	0.9771	0.4541	1.0000
	t-Test p =	0.1241	0.0167	0.0687	0.1091	0.1290	0.9984	0.3841	0.5226	0.3170

Suppl. Table 3. Community State Types (CST) according to VALENCIA

sub-CST	n	CST	n
endometrial			
I-A	29	I	37
I-B	8		
II	5	II	5
III-A	8	III	13
III-B	5		
IV-A	0		
IV-B	2		
IV-C0	0		
IV-C1	2	IV	9
IV-C2	0		
IV-C3	3		
IV-C4	2		
V	7	V	7
total n	71		71

sub-CST	n	CST	n
vaginal			
I-A	31	I	38
I-B	7		
II	6	II	6
III-A	8	III	13
III-B	5		
IV-A	0		
IV-B	2		
IV-C0	0		
IV-C1	3	IV	7
IV-C2	0		
IV-C3	2		
IV-C4	0		
V	7	V	7
total n	71		71

Legend: Classification of endometrial and vaginal samples from 71 patients sequenced with V1-V2 16s rRNA according to https://github.com/ravel-lab/VALENCIA. CST (Community State Types) I – V; sub-CST: additional sub-classifications.

Suppl. Table 4 Comparison of CST-classification for vaginal and endometrial samples

		endometrial				
	CST	I	II	III	IV	V
vaginal	I	37			1	
	II		5		1	
	III			13		
	IV				7	
	V					7

Legend: Classification of endometrial and vaginal samples from 71 patients sequenced with V1-V2 16s rRNA according to https://github.com/ravel-lab/VALENCIA. CST (Community State Types) I – V
